# Confronting Co-workers: Role Models, Attitudes, Expectations, and Perceived Behavioral Control as Predictors of Employee Voice in the Military

**DOI:** 10.3389/fpsyg.2018.02515

**Published:** 2018-12-18

**Authors:** Femke Hilverda, Rick van Gils, Miriam Carla de Graaff

**Affiliations:** ^1^Athena Institute for Research on Innovation and Communication in Health and Life Sciences, VU University Amsterdam, Amsterdam, Netherlands; ^2^Department of Work and Organizational Psychology, Radboud University Nijmegen, Nijmegen, Netherlands; ^3^Ministry of Defense, Hague, Netherlands

**Keywords:** voice, hierarchical organization, organizational performance, undesirable behavior, theory of planned behavior

## Abstract

Speaking up and confronting co-workers when they behave undesirably is important for the well-being of the personnel and organizational performance. In some organizations, a culture of silence prevails, however. Although a number of organizational environments are particularly receptive to employee voice, others are less open to voice behavior, which gives rise to a risk of undesirable behavior. Direct communication (voice) can reduce this enhanced risk. In this study, we used the Theory of Planned Behavior to examine the extent to which attitude, social norm and perceived behavioral control determine voice in hierarchical contexts, which, in general, tend to inhibit voice behavior. For this purpose, a survey study was conducted among military and civilian personnel of the Netherlands Ministry of Defense (*n* = 374). Results showed that employee voice is rather high, regardless of rank, position or gender. Structural equation modeling showed that voice was significantly predicted by perceived behavioral control and injunctive norms (i.e., what is considered to be *normal* in a certain working-environment). Contrary to expectations, voice was not predicted by attitude and descriptive social norms (i.e., what people see that others are *doing* in this respect). Stimulating *confronting* skills and creating a climate in which speaking up is perceived as normal may be beneficial for organizations in general and hierarchical organizations in particular.

## Introduction

Speaking up, confronting co-workers, and communicating expectations and perceptions of undesired behavior are important to team and organizational performance (Qi and Liu, 2017), personnel well-being and organizational survival (Cusack, 2009). Nevertheless, some employees stay silent, whereas others use their voice to speak up when confronted with an undesirable situation or behavior.

In academic literature, *employee voice* refers to the method used by individual employees to (attempt to) improve the situation and solve problems by expressing one’s own opinions and feelings (e.g., Qi and Liu, 2017). Several types of voice have been addressed in the literature, such as *whistle-blowing* (e.g., Loyens, 2013) and prosocial voice. Whistle-blowing focusses on reporting misconduct to an authority who is not the actor, e.g., reporting fraud by peers to a work supervisor or, outside the work environment, providing information to journalists (Loyens, 2013). Formal governance mechanisms, such as complaint systems and regulations, were constructed to stimulate whistle-blowing (Verhezen, 2010). In contrast to whistle-blowing, prosocial voice is a form of voice that is particularly used in working situations between peers, when constructive suggestions are made to improve the situation (Van Dyne et al., 2003; Klaas et al., 2012; Liang et al., 2012). Prosocial voice is an informal governance process, dependent on the resourcefulness of the human capital within the organization in terms of employees’ willingness to improve, high trust and collaborative culture (Verhezen, 2010).

Prosocial voice (hereinafter: *voice*) is the focus of this study. Following Morrison (2011) and Morrison and Milliken (2000), we define voice as the informal communication of opinions, ideas or concerns between direct co-workers, focusing on what has happened in the past with the aim of improving a future situation. Voice is useful as it helps to address and tackle problems in the work environment (Detert and Burris, 2007; Tangirala and Ramanujam, 2008; Qi and Liu, 2017). Employees are often hesitant to speak up (Milliken et al., 2003; Perlow and Williams, 2003) as doing so may challenge the status quo (Liu et al., 2010) and involves a certain degree of personal risk (e.g., perceived risk of losing your job, damaging the relationship with your boss/co-workers). In some organizations, one can even speak of a *culture of silence* among employees (Pinder and Harlos, 2001). That often leads to situations in which misbehavior is not addressed or reported. Certain environments are more susceptible to voice than others. Hierarchy may inhibit voice as individuals have a fear of reprisal. This is also referred to as *injustice-induced silence* (Pinder and Harlos, 2001).

Interestingly, research into voice has focused primarily on behavior directed toward optimization of organizational processes (Van Dyne et al., 2003; Botero and Van Dyne, 2009), with the use of voice in relation to the behavior of co-workers – either unethical or undesirable in some other way – in social interactions largely remaining overlooked. This in spite of the fact that social interactions and (un)ethical behavior toward others are an important aspect of daily job routines and influence team and individual performance within an organizational context. Think, for example, of how bullying negatively influences job satisfaction and how organizational reputation is damaged by fraud and corruption.

When the risk of undesirable behavior is high, it is important that organizational standards are visible in the daily working environment (Fritz et al., 1999). Through direct communication between employees of the same hierarchical level, awareness is created about workplace integrity standards (Fritz et al., 1999). In addition, talking to each other is the most efficient way of exchanging information (Dulye, 2006) and most preferred by individuals when they want to share information on risks (Hilverda and Kuttschreuter, 2018). In this way, voice can prevent unwanted behavior by providing clarity about the desired behavior in a certain situation.

With speaking up in the face of unethical conduct by co-workers at the same hierarchical level having been somewhat overlooked in research into team behavior to date, we believe that it is important to explore voice (intervening by verbally speaking up) in such situations. The military environment exhibits a number of organizational features that give rise to complexity and extremity in interpersonal communication, in both one-to-one and group-level communications. For that reason, we consider the military environment to be ideal for exploring mechanisms of employee voice that can be translated to other organizations that need to cope with similar (ethical) issues albeit in less extreme and less hierarchical environments.

This study addresses the intention of Dutch military and civil servants of the Ministry of Defense to confront co-workers who behave in an undesired manner. In this study, unethical behaviors include both mistreatments – such as (sexual) harassment or intimidation, aggression, stalking, bullying, and discrimination – and non-compliance – such as fraud and conflict of interests.

## Theoretical Background

### Pros and Cons of Voice at the Workplace

Earlier research conducted in an acute care organization provided evidence for the role of peer feedback in encouraging the development of a safety culture and in stimulating initiatives to improve quality (LeClair-Smith et al., 2016). Employee voice also has an essential role to play in effective problem-solving, better decision-making by supervisors and organizational learning (Morrison and Milliken, 2000; Detert and Burris, 2007).

Nevertheless, an employee can be at risk by uttering voice. Speaking up and confronting a co-worker may, for example, damage interpersonal relations (Van Dyne and LePine, 1998). For example, a manager may think of the subordinate as a cynical, not engaged individual. In those situations, the employee runs the risk of receiving a lower performance evaluation, with a higher resultant likelihood of being dismissed (Burris et al., 2013). The literature suggests, however, that cynicism is a self-defense mechanism that helps highly engaged employees to cope with disappointment about the organization and its personnel (Naus et al., 2007). Organizational cynicism can thus be regarded as (the result of) ineffective voice, as the message has not been heard and desired changes have not been carried out. This may even result in more disappointment as misattribution of cynicism to non-involvement rather than voice causes misunderstanding between the employee, the leadership and other team-members. Speaking up may also be disruptive and costly for teams when it challenges the existing conditions too much (MacKenzie et al., 2011).

### Military Culture and (Un)Ethical Behavior

The armed forces is a unique organization in which personnel experience a strong sense of tradition and hierarchy (Andriessen et al., 2017). Tradition and hierarchy increase the risk of undesirable behavior (Andriessen et al., 2017). Moreover, undesirable behavior and mistreatment have not always been reported immediately in military organizations. For example, in the Canadian Armed Forces in the late 1990s, a major scandal only came to light when allegations of harassment and abuse of female soldiers by their fellow (sometimes superior) service members were made public after a long period of silence (Pinder and Harlos, 2001). In contrast, when mistreatment is not directed toward oneself, but is observed as being directed toward others, for example toward the local population in the mission area, one may expect government officials and especially soldiers to intervene, as it is their duty to bring about safety and security. Studies have shown, however, that military personnel may not intervene, speak up or report fellow servicemen who cross a line in, for example, treatment of non-combatants during military operations and deployments (e.g., the Abu Ghraib prison abuses described by Bartone, 2010). For example, a United States assessment of well-being of soldiers deployed to Iraq showed that fewer than 50% of the troops were willing to report a member of their unit for ethical violations (Warner et al., 2011). Due to the high stakes of military operations, for both the soldiers themselves, as well as for the operation and the local population, it is especially important for unethical behavior in the military to be actively prevented and dealt with (De Graaff et al., 2016, 2017). We argue that creating a culture of voice starts in the barracks at the home base in order for this culture to be solidly present in the more extreme and complex situations of deployment.

### Predictors of Voice

To explore the predictors of voice behavior within a military organization, we used the Theory of Planned Behavior (Ajzen, 1991) as a theoretical framework. This theory was developed to explain individuals’ behavior. To our best knowledge, however, it has not been used to explain voice in a military work environment. The Theory of Planned Behavior posits that the intention to perform a certain behavior is the most important predictor of the behavior itself. The intention itself is in turn predicted by attitude toward the behavior, social norms and perceived behavioral control. We aimed to answer the following research question: *To what extent do attitude, social norm and perceived behavioral control predict the intention of speaking up to co-workers who behave undesirably?*

According to the Theory of Planned Behavior, *attitude* determines behavior (Ajzen, 1991; Fishbein and Ajzen, 2011). With respect to speaking up, the attitude consists of two components: outcome expectations (i.e., the expected consequences of speaking up against the undesirable behavior of co-workers) and some degree of inclination or disinclinations with respect to these expectations. Based on the Theory of Planned Behavior, a positive relationship between attitude and voice was expected: The more positive the attitude, the higher the intention to speak up (H1).

The Theory of Planned Behavior also emphasizes the importance of *social norm* on behavior. Social norms reflect the perceived expectations in the social environment (Tankard and Paluck, 2016). They consist of the normative beliefs and the motivation to conform to these beliefs (Ajzen, 1991; Fishbein and Ajzen, 2011). Social norms can be divided into injunctive norms and descriptive norms, both focused on the perception of the individual at stake (Rimal and Real, 2003). While injunctive norms refer to the extent to which individuals feel pressured into engaging in a particular behavior, descriptive norms refer to the individual’s beliefs about how widespread the behavior is among a particular referent group (Rimal and Real, 2003; van den Bongardt et al., 2014). Injunctive norms therefore encompass the perceived consensus about acceptable behavior, whereas descriptive norms refer to the individual’s perception of what others are *doing* (Dixon et al., 2015).

Research showed that social norms are important in a military context. Firing et al. (2012) showed the importance of descriptive norms in relation to obedience. In their study, Norwegian cadets were ordered to jump into cold seawater as part of a compulsory military training. When a peer made the suggestion not to jump, cadets were more hesitant to jump. Without this suggestion, 76.3% of the cadets jumped into the cold water; with this suggestion, only 51.4% of the cadets jumped. It was thus predicted that both injunctive norms (H2a) and descriptive norms (H2b) have a positive relationship with voice. The higher the perceived norms in favor of voice, the higher the intention to speak up when a co-worker acts inappropriately.

The final construct in the Theory of Planned Behavior is *perceived behavioral control*. Perceived behavioral control refers to the degree to which someone considers himself/herself capable of performing a certain behavior (Ajzen, 1991; Fishbein and Ajzen, 2011). Within a military context, perceived behavioral control has been found to have an effect on behavior. Laudenslager et al. (2004) showed that members of the United States Air Force had a higher intention to act in an environment friendly way (recycle and save energy) when they experienced higher levels of perceived behavioral control. Based on this, we hypothesized a positive relationship between perceived behavioral control and voice: the higher employees score on perceived behavioral control, the higher the intention to speak up (H3).

## Materials and Methods

### Participants and Procedure

A survey was conducted among the personnel of the Netherlands Ministry of Defense in April and May 2017. Participants were recruited via e-mail, internal communication outlets, and social media. Participants were requested to fill out an online questionnaire, which took about 15 min to complete. A total of 10 participants filled out a hard-copy version of the survey as they did not have access to the Internet.

This study was carried out in accordance with the ethical guidelines of the Behavioral Faculty of Radboud University with written (digital) informed consent from all subjects in accordance with the regulations of the principles of the Declaration of Helsinki. This study did not require ethical approval according to the Dutch WMO law (Medical Research Involving Human Participants Law), because it did not include experimental data of patients and consisted of an online survey. Participants were allowed to withdraw from the study at any time without proving a reason and gave informed consent before starting the survey. The data of the participants was anonymized in a way that it could not be traced back to any of the participants.

A total of 554 respondents started the survey. Approximately 67.5% completely filled out the survey and were found eligible for the purpose of our study (i.e., they were employed at the Ministry of Defense) and were subsequently included in our analysis. This research sample consisted of 374 respondents who were employed at the Netherlands Ministry of Defense. The sample consisted of 243 males (65.0%), 130 females (34.8%), and one respondent who indicated “other” as gender. Distribution across age categories was as follows: 59 participants were aged between 21 and 30 years old (15.8%), 97 participants were aged between 31 and 40 years old (25.9%), 107 participants were aged between 41 and 50 years old (28.6%), 99 participants were aged between 51 and 60 years old (26.5%), and only 12 participants were older than 60 years old (3.2%). A total of 40.4% of respondents were in a leadership position. While that might seem to be a large proportion, it should be borne in mind that the leadership starts at the level of corporal in the Dutch military organization. A small majority was working as active military personnel (52.1%; *n* = 195), while the other respondents were civilian employees, which is representative for the Ministry of Defense in 2018 (Ministry of Defense, 2018).

### Measurement

The survey was partly based on explorative interviews that were conducted in March 2017. A pilot study was performed using a small sample of employees (n = 6) to examine clarity of the items. After the pilot study, several items were slightly reformulated in order to make them easier to understand for the respondents. A table with scales, reliability, and items per construct is added in Appendix A.

#### Voice

Employee voice was measured by three items (α = 0.89). Respondents indicated on a 7-point Likert scale (from *1 = strongly disagree* to *7 = strongly agree*) the extent to which they were inclined to speak up against a co-worker who behaves undesirably in the coming month.

#### Attitude

Attitude was measured by outcomes expectations (α = 0.80) and an evaluation of these expected consequences of speaking up (α = 0.82). Based on the explorative interviews, six expected outcomes were defined: the colleague will learn from his behavior, the colleague will be hurt, the cooperation will improve, mutual trust will grow, the actor will experience negative consequences later on, and the colleague will think negatively about the actor. Respondents indicated on a 7-point Likert scale the extent to which they agreed that voice would have these outcomes (from *1 = strongly disagree* to *7 = strongly agree*) and evaluated the outcomes (from *1 = very negative* to *7 = very positive*). Scores on negative outcomes were recoded. Confirmatory Factor Analysis (CFA; see results section) indicated that these negatively formulated items fitted poorly on the construct attitude and were consequently excluded from further analysis. Product scores per item were used in the analysis.

#### Social Norms

Based on the explorative interviews, five referent groups were defined: subordinates in rank or in pay scale; peers (immediate co-workers); direct supervisor(s); indirect supervisor(s); and the highest management layer in the defense department. Injunctive norms were measured by five items. Respondents indicated on a 7-point Likert scale (from *1 = strongly disagree* to *7 = strongly agree*) the extent to which they agreed that each of the referents expected them to speak up in the event of undesirable behavior by co-workers (α = 0.87). Descriptive norms (α = 0.85) were measured in a similar vein. Respondents were requested to indicate on a 7-point Likert scale (from *1 = never* to *7 = always*) how often each of the referents spoke up to their colleagues.

#### Perceived Behavioral Control

Perceived behavioral control was measured by five items (α = 0.80). Respondents indicated on a 7-point Likert scale (from *1 = strongly disagree* to *7 = strongly agree*) the extent to which they agreed that they were capable of speaking up against a co-worker who behaved undesirably.

#### Common Method Bias

A possible limitation in self-reported survey studies is *common method bias*, as data on the different constructs, both independent variables and dependent variable, are collected with the same instrument at the same time of measurement (Podsakoff et al., 2003). Therefore, Harman’s single factor test was performed in SPSS to explore whether using the same method was problematic in our study. This test showed that the common variance was approximately <29%, which is under the threshold of 50% recommended by Lowry and Gaskin (2014). This indicates that the variance in the data is not likely due to having used a single method.

### Analysis

First, Confirmatory Factor Analyses (CFA) in AMOS were performed to ensure convergent validity and discriminant validity for each of the constructs. Error terms of items within the same constructs were allowed to correlate. Convergent validity, i.e., items are related to their predicted construct rather than other constructs, was assessed on the basis of CFA-factor loadings, Cronbach’s alpha and average variances extracted (Hair et al., 2006). Discriminant validity, i.e., constructs are measuring different concepts, was assessed by comparing the Pearson correlations between all the constructs computed in SPSS with the square root of the AVEs obtained in AMOS.

To get insight in the data means, standard deviation and correlations between construct were calculated. After that, exploratory ANOVAs were conducted to test for differences between gender (male/female), military personnel versus civilian personnel and leadership role (yes/no) in intention to confront co-workers who behave unethically. If significant differences were found, those characteristics were used in a multigroup analysis to examine possible confounding effects.

Structural equation modeling (SEM) using AMOS was applied to test the model of determinants of voice. Voice was used as the dependent variable. Attitude, injunctive and descriptive subjective norms, and perceived behavioral control were included as predictors. The constructs were included as latent variables in the model using the single items as indicators. Attitude was one exception where product scores were used as indicators instead of item scores.

Using the most commonly used criteria (Raykov et al., 1991) model fit was considered to be good when the Root Mean Square Error of Approximation (RMSEA) was below 0.08 (Kline, 2005), the normed chi-square was below 3.00 (Bollen, 1989; Kline, 2005), the Comparative-Fit Index (CFI), Tucker-Lewis Index (TLI) were above 0.90 (Bollen, 1989; Marcoulides and Schumacker, 2013). In addition, the chi-square statistic should be non-significant and the value of the goodness-of-fit index (GFI), a transformation of the chi-square, should be above the acceptability threshold of 0.90 (Kline, 2005). As these last two measures are highly dependent on sample size and model size (Fornell and Larcker, 1981), we considered them to be less applicable to our study.

## Results

### Confirmatory Factor Analyses

Confirmatory Factor Analyses showed acceptable measurement of voice, injunctive norms, descriptive norms and perceived behavioral control. All factor loadings exceeded the threshold for importance of 0.40 (Hair et al., 2006), indicating that the factors (i.e., latent constructs) at least explain 16% of variance in item responses. Reliability was no lower than 0.80, which is higher than the threshold of 0.70. Average variance extracted ranged between 0.47 and 0.74, with descriptive norms (0.49) and perceived behavioral control (0.47) just below the threshold of 0.50, implying sufficient convergent validity (Fornell and Larcker, 1981).

The measurement of attitude was problematic, however. Three out of six items had very low factor loadings (0.09; 0.12; 0.19). It was therefore decided to exclude these items from our analyses. After deletion of these items, convergent validity was good, with factor loadings above 0.68, reliability of 0.87, and AVE of 0.72.

With regard to discriminant validity, the correlations between the constructs were all smaller than the square roots of AVE. This means that discriminant validity was good (Chin, 1998).

### Means and Correlations

Table 1 describes the means, standard deviation and correlations of the constructs. Voice was rather high (*M* = 5.28), and so were perceived behavioral control (*M* = 5.33) and injunctive norms (*M* = 5.40). In contrast to that, the descriptive norms were a bit lower (*M* = 3.66), just below the midpoint of the scale. Attitude was computed as a product score ranging from 1 to 49. The mean of 24.09 was somewhat around the midpoint of that scale.

**Table 1:** Mean, standard deviations, and correlations of the constructs (*N* = 374).

Constructs		Mean	sd	Correlations				
				**1**	**2**	**3**	**4**	**5**
1	Voice	5.28	1.36	1					
2	Attitude	24.09	9.28	0.37^∗∗^	1				
3	Injunctive norms	5.40	1.26	0.44^∗∗^	0.31^∗∗^	1			
4	Descriptive norms	3.66	1.24	0.35^∗∗^	0.35^∗∗^	0.42^∗∗^	1		
5	Perceived behavioral control	5.33	1.09	0.65^∗∗^	0.41^∗∗^	0.42^∗∗^	0.32^∗∗^	1	

*^∗∗^Correlation is significant at the 0.01 level (2-tailed). Attitude scores are product scores per item that were summed (possible scores range between 3 and 147) and then averaged ranging from 1 to 49. Product scores were used in the model analysis. All other constructs were scored on a 7-point scale. Item scores were used in the model analysis.*

All correlations were significant. Voice was most strongly related to perceived behavioral control (*r* = 0.65), followed by injunctive norms (*r* = 0.44). The higher the perceived behavioral control and perceived norms, the higher the intention to confront a co-worker. Descriptive norms and attitude seemed to be less important.

Three separate ANOVAs were conducted to test for differences between gender groups (male/female), military versus civilian personnel and leadership role (yes/no) in voice. Results indicated that, although men had a higher intention to confront (*M* = 5.39, *SD* = 1.38) than women (*M* = 5.11, *SD* = 1.30), this difference was not significant, *F*(1,371) = 3.70, *p* = 0.055. A significant difference on voice, however, was found between military (*M* = 5.48, *SD* = 1.24) and civilian personnel (*M* = 5.06, *SD* = 1.44), *F*(1,372) = 9.16, *p* = 0.003. In addition, personnel in leadership positions (*M* = 5.74, *SD* = 1.12) showed a significantly higher intention to confront compared to personnel for whom leadership was not part of their working activities (*M* = 4.97, *SD* = 1.42), *F*(1,372) = 31.05, *p* < 0.001.

### Structural Equation Modeling

**FIGURE 1:**
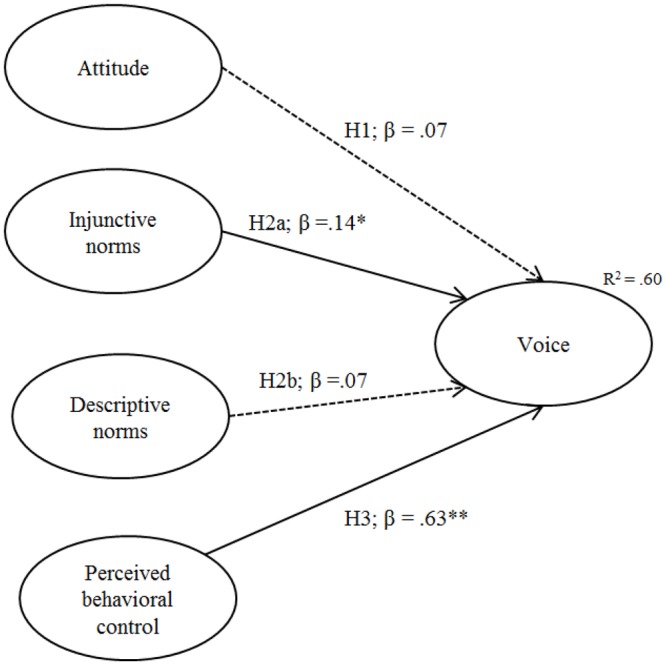
Predictors of voice. ^∗∗^*p* < 0.01, ^∗^*p* < 0.05. Solid arrow: significant path, dashed arrow: insignificant path.

The structural model was tested based on the Theory of Planned Behavior. Figure 1 shows the model tested. The RMSEA (0.052), normed chi-square (2.02), CFI (0.96), and TLI (0.96) all indicated a good model fit. In addition, the GFI was 0.92, indicating a good model fit. However, the chi-square statistic, χ^2^(175) = 352.72, was significant. As this measure is highly dependent on sample size and model size, we still considered the model fit to be good (Fornell and Larcker, 1981).

The model explained 60% of the variance in voice behavior. Looking at the individual effects of the determinants, it is shown that perceived behavioral control had the largest significant effect on voice (β = 0.63, *p* < 0.001). This means that the more able participants perceived themselves to be with respect to speaking up to co-workers, the higher their intention to do so was. In addition, injunctive social norms had a significant effect (β = 0.14, *p* = 0.01), implying that voice behavior is determined by the perceived expectations of the social environment. In contrast, there was no significant relationship between descriptive norms and voice or attitude and voice (β = 0.07, *p* = 0.16, and β = 0.07, *p* < 0.14, respectively). With respect to the covariance between exogenous predictors, there were no indications for mediating or moderating effects.

### Additional Analysis: Multigroup Analysis

Based on the difference in voice behavior found between employees with a leadership position and those who were not in such a position on voice, a multi-group analysis was performed in AMOS splitting up employees with a leading position and those who were not in such position. First, the unconstraint model was tested without any constraints. After that, a model with four constraints was tested: the regression weights of the predictors were set to be equal in both groups. The difference between these two models (Δ*χ^2^* = 6.49, df = 4, *p* = 0.17) was not significant, implying that leadership did not have a moderating role in our model.

The same procedure was applied for military versus civilian personnel. In the unconstraint models, descriptive norms (and perceived behavioral control) were significant predictors for military personnel, whereas injunctive norms (and perceived behavioral control) were significant predictors for civilian personnel. In spite of this difference in significant predictors, there was no significant difference between the unconstraint model and the constraint model in which the regression weights of the predictors were set to be equal (Δ*χ^2^* = 2.38, df = 4, *p* = 0.67). This implies that one’s status as military versus civilian personnel is not a moderator in our model. The results of the constraint model are comparable to the full structural model as described in Figure 1.

## Discussion

### Discussion of the Results

In this study, we explored the intention of employees of The Ministry of Defense in the Netherlands to confront co-workers when they behave in an undesired manner, specifically in social interactions. The Theory of Planned Behavior was used as a theoretical framework to answer the following research question: *To what extent do attitude, social norm, and perceived behavioral control predict the intention of speaking up to co-workers who behave undesirably?*

Overall, the results showed that voice is rather high, regardless of rank, position or gender. Leaders showed a significantly higher intention to confront co-workers than non-leaders, while military personnel showed a higher intention compared to civilian personnel. The significant difference between leaders and non-leaders might be explained by the fact that leaders are (on the basis of their role) held accountable/responsible for all kinds of actions of (individual) team members. As such, they are experienced and trained in effectively confronting others as part of their day-to-day jobs. This experience and training in confrontation may enhance perceived behavioral control, which in turn makes it easier for them to speak up to others at similar levels as their own (peers) when they feel personally mistreated. Multi-group analysis, however, showed that leadership was not a moderator in our model. This means that, though leaders have a higher intention to speak up compared to non-leaders, the relationships between the independent variables and dependent variable were the same across groups.

This brings us to the predictors that were found to be relevant for voice. The total model explained 60% in variance in voice and therefore the Theory of Planned Behavior proved to be useful for examining this type of behavior in a military context. Perceived behavioral control (H3), and injunctive norms (H2a) significantly predicted the intention to confront, showing a positive relationship with voice. Attitude (H1) and descriptive norms (H2b) were not significantly related to voice.

Perceived behavioral control turned out to be the strongest positive predictor of voice. When defense employees experience higher levels of behavioral control, meaning they consider themselves capable of speaking up and able to positively influence the consequences of confronting, the intention to confront others for undesirable behavior is higher. This is in line with previous research (Rich et al., 2015). Results showed that perceived behavioral control was high in our sample.

The results showed that the second important predictor for voice was injunctive social norms. Perceiving that others expect you to confront, leads to an increase in the intention to confront. This is in line with the existing literature (Ajzen, 1991; Fishbein and Ajzen, 2011; van den Bongardt et al., 2014). Strikingly, the descriptive social norms did not predict voice in the current research. This means that seeing others setting a *good example* in regards to confronting others did not predict the intention to confront. This is inconsistent with previous studies (e.g., Rivis and Sheeran, 2003). One possible explanation might be that actually confronting another individual is often a rather private action, meaning that *third parties* do not see this behavior actually taking place. As the injunctive norms are an important predictor for voice (and scored rather high), it is probable that colleagues do share their ideas about the relevance of speaking up (their reflections and evaluations) to other colleagues (as well as third parties).

Interestingly, attitude did not show a significant effect. The outcomes that were related to voice behavior and that were used to create the attitude items were based on initial interviews. Although some of these outcomes loaded very well on the attitude factor, some others did not. This problem regarding the measurement of attitude might have prevented us from detecting the effect of attitude on voice. This implies that more research is needed into the perceived consequences of voice. While the positive outcomes seem to form a coherent factor, the negative consequences were perceived differently by the respondents.

Results from this study can be used to develop interventions to create a psychologically safe working environment. They are therefore relevant to the Netherlands Ministry of Defense, as well as being very relevant to other organizations. It is essential to stimulate other-oriented behavioral motives underlying voice that are based on cooperation feeling and altruism to enhance prosocial voice, a type of voice that is both constructive for individual employees as well as for the organization (Van Dyne et al., 2003; Walumbwa and Schaubroeck, 2009).

### Limitations and Future Research

This exploratory research showed the value of the Theory of Planned Behavior in explaining voice in a military context. Though this theory was useful in giving first insights in the determinants of voice, other determinants might be relevant as well for obtaining a more complete picture of voice in military contexts. For example, job satisfaction and detachment (i.e., the intention to leave the organization) are suggested as important predictors of the intention to confront (Klaas et al., 2012). Employees with a high level of job satisfaction are more proactive (Strauss et al., 2015), which may lead to higher intentions to confront co-workers. Also (ethical) leadership and work-group psychological safety are considered to be relevant influencers of employee voice behavior (Walumbwa and Schaubroeck, 2009). Furthermore, psychological strain may have an influence on voice as research showed that psychological strain partially mediated the relation between learning climate and performance (Cortini et al., 2016). In a similar vein, psychological strain might impact the relation between social norms and voice behavior.

Another point to consider for further research is the focus on behavior instead of intention. As we used a survey study, we measured voice intentions rather than voice behavior. Follow-up research may take this one step further when investigating employee voice behavior in real life settings. In addition, future research should include different types of measurement to prevent the occurrence of common method bias. It would also be interesting to address how online environments are used as an outlet for voice within organizations, as research shows the increasing opportunities of social media for interpersonal interactions. For example, research has shown that social media–mediated interaction with similar others induced sense-making behavior (Hilverda et al., 2017), which might support the value of social media for voice behavior. While social media might help employees in this way, they are often unware of the risk of doocing that goes hand in hand with it (Cortini and Fantinelli, 2018).

### Organizational Recommendations

To encourage voice, we consider two main recommendations to be relevant for organizations in general. First, it is important for employees to feel capable of speaking up. That is, they need both personal competency and no or few processes that make it difficult to confront others. For example, in the Netherlands it is quite common in current organizational design to implement alternative workplace strategies, which involves employees working at their own pace at variable locations or in open-plan offices. As such, contact between team members is less direct and when it is direct it is less private. Thus, when a colleague behaves inappropriately it becomes more difficult to confront him/her because private meetings rarely take place. That reduces the degree of perceived safety as it is uncertain how the other will respond: how large are the risks of negative consequences for the actor? Meetings are infrequent and other employees are constantly around. Such alternative workplace strategies might thus be hindering voice and demand an alternative approach to stimulate voice when it is not naturally present, for example by creating specific feedback sessions for employees.

Second, as the results indicate, it is vital to create a working environment in which employees consider it normal to speak up when they feel mistreated by colleagues’ undesired or unethical behavior. In order to establish such a normalcy, it is important that a dialog about the relevance and necessity of voice is present within the organization, and that employees do not perceive the measures and reactions of the other and/or the management as harmful. Explicitly stating that voice behavior is wanted and necessary is important here. In organizations like the military, where the stakes are high, it is quite common to think of error management in terms of error prevention. Though error prevention seems positive, it often leads to negative consequences, such hiding errors rather than adequately dealing with them (Dimitrova et al., 2016). Creating an environment in which errors can be addressed without negative consequences is important. Think for example of crew resource management and the learning climate in civil aviation and hospital settings (Haerkens et al., 2012; Cortini et al., 2016).

### Conclusion

Following the exploratory results of this study, it can be said that, in our study, voice intentions are mostly influenced by perceived behavioral control (“can I do it?”) and to a lesser extent injunctive norms (“do I feel pressure to do it?”). Attitudes and descriptive norms do not play an important role. We therefore argue that a safety culture is a perfect breeding ground for making the move to voice in interpersonal interactions. After all, when it is quite common for the nurse to make suggestions and point out possible risks and failures to a doctor during a medical procedure, or for the flight engineer to overrule the pilot when it comes to safety regulations, then why not speak up and confront when the nurse or the engineer feels disrespected and hurt by gossip, intimidation or bullying in the same context?

## Author Contributions

RvG performed this study as part of his masters’ program at Radboud University, Nijmegen, Netherlands. The study was supervised by FH, who was at that point working at Radboud University as a lecturer and MdG (Ministry of Defense). FH performed statistical analyses, interpreted data, drafted the manuscript, and acted as corresponding author. MdG provided a critical overview of the project and edited the manuscript. All authors read and approved the final version of the manuscript.

## Conflict of Interest Statement

The authors declare that the research was conducted in the absence of any commercial or financial relationships that could be construed as a potential conflict of interest.

## Appendix

**APPENDIX A:** Scales, items and reliabilities of constructs (*n* = 374).

Measures	Characteristics	
	Scale	Reliability
1.*Voice*		
(1) When I observe undesirable behavior from an immediate co-worker over the next month, I intend to confront him/her	7-point Likert scale from *1 = strongly disagree* to *7 = strongly agree*	0.89
(2) I am sure that I would confront an immediate co-worker if I were to witness undesirable behavior by him/her in the coming month
(3) I will try to confront an immediate co-worker whom I observe behaving in an undesirable way over the next month
2.*Attitude Outcome expectations:*
Confronting a co-worker when he/she behaves undesirable, has the following consequence…	7-point Likert scale from 1 = *strongly disagree* to 7 = *strongly agree*	0.80
(1) The colleague will learn from the behavior
(2) The colleague will be hurt[R]
(3) The cooperation will improve
(4) Mutual trust will grow
(5) I will experience negative consequences later on[R]
(6) The colleague will think negatively about me[R]
*Evaluation:*
How positive are the following possible consequences of confronting a co-worker when he/she behaves undesirably ?	7-point Likert scale from *1 = very negative* to *7 = very positive*	0.82
(1) The colleague will learn from his behavior
(2) The colleague will be hurt[R]
(3) The cooperation will improve
(4) Mutual trust will grow
(5) I will experience negative consequences later on[R]
(6) The colleague will think negatively about me[R]
3. *Social norm*
Descriptive norm:
To what extent do the following people expect that you confront your co-workers?	7-point Likert scale from 1 = *strongly disagree* to 7 = *strongly agree*	0.87
(1) Your subordinates in rank or in pay scale
(2) Your peers (immediate co-workers)
(3) Your direct supervisor(s)
(4) Your indirect supervisor(s)
(5) The highest management layer in the defense department
*Injunctive norm:*
In your opinion, how frequently do the following person(s) confront their immediate co-workers?	7-point Likert scale from *1 = never* to *7 = always*	0.85
(1) Your subordinates in rank or in pay scale
(2) Your peers (immediate co-workers)
(3) Your direct supervisor(s)
(4) Your indirect supervisor(s)
(5) The highest management layer in the defense department
4. *Perceived behavioral control*
(1) There is nothing preventing me from confronting an immediate co-worker if I feel the need to do so	7-point Likert scale from 1 = *strongly disagree* to 7 = *strongly agree*	0.80
(2) I have all the skills I need to confront an immediate co-worker with his/her undesirable behavior
(3) I have full control over the choice of whether to confront an immediate co-worker with his/her undesirable behavior
(4) When I experience undesirable behavior and want to address it, it is important for me to see myself as being capable of confronting a co-worker with his/her undesirable behavior.
(5) If I observe undesirable behavior, I am willing to accept the risk of experiencing negative consequences of confronting the immediate co-worker.

*[R] = negatively formulated items (recoded). Based on the results of a Confirmatory Factor Analysis, it was decided not to use these items in the subsequent analyses.*
